# Supplementary material for: Transcriptome Analysis Unravels Key Factors Involved in Response to Potassium Deficiency and Feedback Regulation of K+ Uptake in Cotton Roots
Source: Int J Mol Sci. 2021 Mar 19;22(6):3133. doi: 10.3390/ijms22063133 (PMC8003395; doi:10.3390/ijms22063133)
Supplement: Supplementary file 1 [file ijms-22-03133-s001.zip › Supplementary Files/2021.03.18 Supplementary Figures.docx]

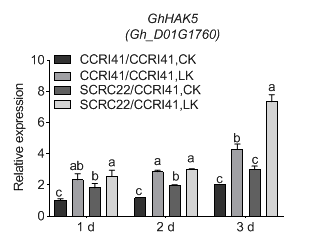


**Figure S1.** Effects of potassium (K^+^) deficiency on the expression of the marker gene *GhHAK5* (*Gh_D01G1760*) in roots of CCRI41/CCRI41 (scion/rootstock) and SCRC22/CCRI41 at the three-leaf stage. Fine roots with diameter less than 1 mm were collected at 1, 2 and 3 d after low K^+^ treatment (LK, 0.03 mM K^+^) for qRT-PCR (quantitative real-time PCR) analysis. Control plants were grown under sufficient K^+^ (CK, 2.5 mM K^+^). The data are shown as means ± SD from three replicates (*n* = 3). Different letters indicated significant differences at 5% level.

**Figure S2.** Comparison of RNA sequencing (RNA-Seq) and quantitative RT-qPCR (RT-qPCR) for randomly selected genes. CR and LR: roots from control (CK, 2.5 mM K^+^) and low K^+^ (LK, 0.03 mM K^+^) treated plants; 41/41: CCRI41/CCRI41 (scion/rootstock); 22/41: SCRC22/CCRI41. Error bars represent the standard deviation following qRT-PCR analysis (*n* = 3).


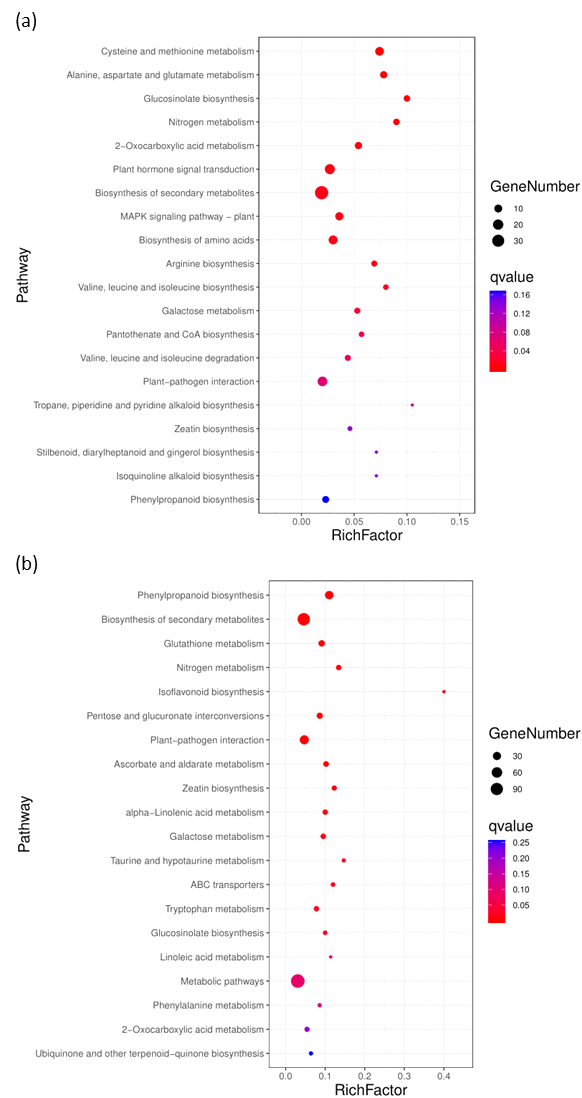


**Figure S3.** Top 20 enriched Kyoto Encyclopedia of Genes and Genomes (KEGG) pathways based on the DEGs in R common (a) and R22/41 unique group (b). R common: the common differentially expressed genes (DEGs) in roots of CCRI41/CCRI41 (scion/rootstock) and SCRC22/CCRI41 under potassium (K^+^) deficiency. R22/41 unique: the DEGs specifically identified in the roots of SCRC22/CCRI41.

**Figure S4.** The Fragments Per Kilobase Million Mapped Reads (FPKM) value of several differentially expressed genes (DEGs) related to potassium (K^+^) transporter and K^+^ channel in R common group and R_22/41_ unique. R common: the common DEGs in the roots of CCRI41/CCRI41 (scion/rootstock) and SCRC22/CCRI41 under K^+^ deficiency. R22/41 unique: the DEGs specifically identified in the roots of SCRC22/CCRI41. CR and LR: roots from control (2.5 mM K^+^) and low K^+^ (0.03 mM K^+^)-treated plants; 41/41: CCRI41/CCRI41; 22/41: SCRC22/CCRI41.


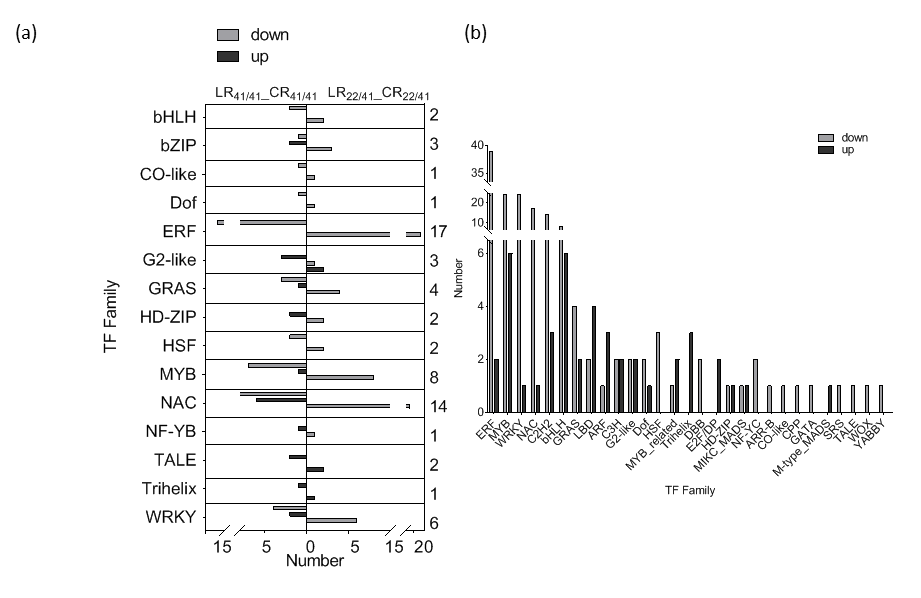


**Figure S5.** The differentially expressed genes (DEGs) related to transcription factors (TFs) in R common (a) and R_22/41_ unique (b) group. R common: the common DEGs in roots of CCRI41/CCRI41 (scion/rootstock) and SCRC22/CCRI41 under potassium (K^+^) deficiency. R22/41 unique: the DEGs specifically identified in the roots of SCRC22/CCRI41. CR and LR: roots from control (2.5 mM K^+^) and low K^+^ (0.03 mM K^+^)-treated plants; 41/41: CCRI41/CCRI41; 22/41: SCRC22/CCRI41.


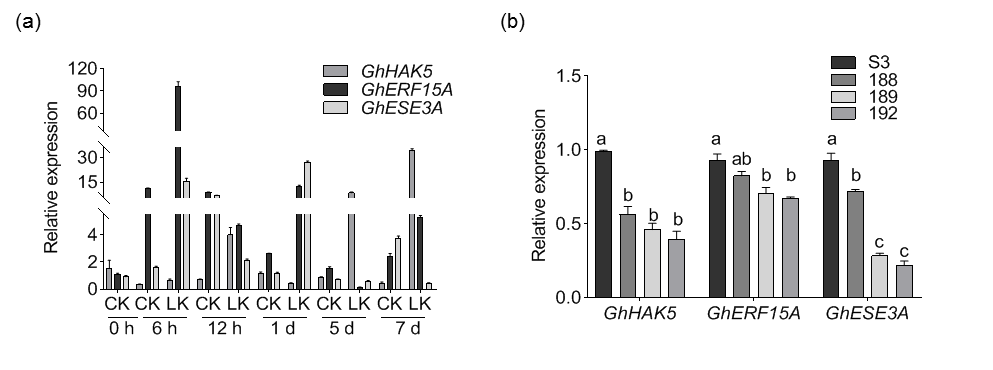


**Figure S6.** The expression pattern of *GhHAK5* (*Gh_D01G1760*)*, GhERF15A* (*Gh_A08G1686*) and *GhESE3A* (*Gh_A07G251*) in cotton roots . (a) Seedlings of SCRC22 were treated with low K^+^ (LK, 0.03 mM) at the three-leaf stage, with 2.5 mM K^+^ as control (CK). (b) The relative expression of *GhHAK5, GhERF15A* and *GhESE3A* in the roots of three-leafed *GhHAK5*-RNAi lines (188, 189, and 192) and wild type (S3).
